# Interventions and approaches to integrating HIV and mental health services: a systematic review

**DOI:** 10.1093/heapol/czw169

**Published:** 2017-11-24

**Authors:** Fiona Leh Hoon Chuah, Victoria Elizabeth Haldane, Francisco Cervero-Liceras, Suan Ee Ong, Louise A Sigfrid, Georgina Murphy, Nicola Watt, Dina Balabanova, Sue Hogarth, Will Maimaris, Laura Otero, Kent Buse, Martin McKee, Peter Piot, Pablo Perel, Helena Legido-Quigley

**Affiliations:** 1 Saw Swee Hock School of Public Health, National University of Singapore, 12 Science Drive 2, #10-01, Tahir Foundation Building, 117549 Singapore; 2 Centre for Tropical Medicine and Global Health, Nuffield Department of Clinical Medicine, University of Oxford, Oxford, UK; 3 The Centre for Health and Social Change (ECOHOST), London School of Hygiene & Tropical Medicine, 15-17 Tavistock Place London, London WC1H 9SH, UK; 4 London School of Hygiene and Tropical Medicine, London WC1H 9SH, UK; 5 Centre for Global Non Communicable Diseases, London School of Hygiene & Tropical Medicine; 6 London Borough of Waltham Forest, UK; 7 Haringey Council, UK; 8 Nursing Section, Faculty of Medicine, Universidad Autónoma de Madrid, Madrid, Spain; 9 CIBER of Epidemiology and Public Health (CIBERESP-ISCIII), Madrid, Spain; 10 The World Heart Federation, Geneva, Switzerland

**Keywords:** HIV, integration, mental health

## Abstract

**Background:**

The frequency in which HIV and AIDS and mental health problems co-exist, and the complex bi-directional relationship between them, highlights the need for effective care models combining services for HIV and mental health. Here, we present a systematic review that synthesizes the literature on interventions and approaches integrating these services.

**Methods:**

This review was part of a larger systematic review on integration of services for HIV and non-communicable diseases. Eligible studies included those that described or evaluated an intervention or approach aimed at integrating HIV and mental health care. We searched multiple databases from inception until October 2015, independently screened articles identified for inclusion, conducted data extraction, and assessed evaluative papers for risk of bias.

**Results:**

Forty-five articles were eligible for this review. We identified three models of integration at the meso and micro levels: single-facility integration, multi-facility integration, and integrated care coordinated by a non-physician case manager. Single-site integration enhances multidisciplinary coordination and reduces access barriers for patients. However, the practicality and cost-effectiveness of providing a full continuum of specialized care on-site for patients with complex needs is arguable. Integration based on a collaborative network of specialized agencies may serve those with multiple co-morbidities but fragmented and poorly coordinated care can pose barriers. Integrated care coordinated by a single case manager can enable continuity of care for patients but requires appropriate training and support for case managers. Involving patients as key actors in facilitating integration within their own treatment plan is a promising approach.

**Conclusion:**

This review identified much diversity in integration models combining HIV and mental health services, which are shown to have potential in yielding positive patient and service delivery outcomes when implemented within appropriate contexts. Our review revealed a lack of research in low- and middle- income countries, and was limited to most studies being descriptive. Overall, studies that seek to evaluate and compare integration models in terms of long-term outcomes and cost-effectiveness are needed, particularly at the health system level and in regions with high HIV and AIDS burden.

## Introduction

Key Messages

- Available literature on interventions integrating HIV and mental health services reveal that there is much diversity in the approaches adopted in combining treatment modalities; ranging from integration within a single facility, to multi-facility integration, and integrated care coordinated by non-physician case managers.
- Existing evidence, although limited, suggest that integrating HIV and mental health services may be linked to improved patient and service delivery outcomes in diverse settings.
- There is a need for higher quality and robustly designed studies to evaluate and compare integration models at different levels of service delivery in terms of long-term impact on patient outcomes and cost-effectiveness, particularly in low- and middle-income countries with high HIV and AIDS burden.

In comparison with the general population, people living with HIV (PLHIV) are more likely to experience mental health disorders such as depression, anxiety, suicidality, and substance misuse (Chibanda *et al*. 2016, Hughes *et al*. 2016, Sherr *et al*. 2011, Clucas *et al*. 2011, Catalan *et al*. 2011, Brandt 2009). In low- and middle-income countries (LMICs), the prevalence of these common mental disorders is over 30% among PLHIVs (Chibanda *et al*. 2014). With estimates of 36.9 million PLHIVs globally, the burden of disease is significant (UNAIDS 2015). In fact, current predictors indicate that both HIV and AIDS, as well as depression will be the first two leading causes of disability globally by 2030 (Pappin *et al*. 2012, Gupta *et al*. 2010).

The association between mental health problems and HIV and AIDS is complex and bi-directional. HIV virus and opportunistic infections associated with AIDS can cause neurological damage (Dube *et al*. 2005), while mental health problems can also arise as a side effect of antiretroviral treatment or from the stigma, stress and socio-economic predicaments associated with the infection and treatment process (Moore *et al*. 1996, Yi *et al*. 2015). On the other hand, depression and substance use disorders, which commonly occur together is known to increase the risk of behaviours that promote HIV transmission, such as risky sexual activity and injecting drug-use (van Empelen *et al*. 2003). International evidence have found that populations with severe mental illness have higher rates of HIV infection (Senn and Carey 2009). Mental illness can also have a detrimental impact on adherence to antiretroviral therapy and progression of AIDS, leading to poorer health outcomes (Buckingham *et al*. 2013). Collectively, the cluster of diagnoses – HIV, mental illness, and substance abuse disorders – has emerged as a distinct clinical condition wherein patients experience a complex set of medical, psychological and social complications that need to be tackled through integrated care. Against this backdrop, many landmark publications including the UNAIDS Strategy 2016-2021 (UNAIDS 2016) and The Grand Challenges in Global Mental Health Initiative (Kaaya *et al*. 2013) have called for a stronger commitment towards integration of HIV and non-communicable diseases including mental illness and drug dependency.

Although the need for integrating HIV and mental health services is indisputable, the challenges are evident in implementing service integration that is cost-effective, and of high quality and impact. In LMICs, health systems are commonly overstretched due to poor human and financial resource, and oriented to treating acute conditions, resulting in fragmented care and poor sustainability of healthcare services for long-term disorders like HIV and mental illness (Semrau *et al*. 2015, Jacob *et al*. 2007). While high-income countries may have health systems that are better able to deal with a relatively lower overall burden of disease, literature from these countries has shown that initiatives which work initially have a tendency to be less effective when scaled-up (Parry *et al*. 2013). For these reasons, it is imperative to form an evidence base on what does and does not work in promoting HIV and mental health service integration.

Previous systematic reviews have examined HIV risk behaviours among adults with severe mental illness (Meade and Sikkema 2005); the link between mistreatment in childhood disorders, mental health disorders, and HIV infection (Spies *et al*. 2012); and literature on HIV and mental illness in low income countries (Collins *et al*. 2006). Studies have also reviewed intervention trials to improve mental health among PLHIVs in LMICs (Sikkema *et al*. 2015); as well as interventions using specific approaches like cognitive-behavioural therapy (Crepaz *et al*. 2008) or that target specific disorders such as depression (Sherr *et al*. 2011), anxiety (Clucas *et al*. 2011) and suicidality (Catalan *et al*. 2011) among PLHIV. A dearth of evaluated mental health services in HIV care is still evident, particularly in LMICs (Kaaya *et al*. 2013). We are unaware of any systematic review of the existing systemic approaches to the integration of mental health and HIV and AIDS services, and their effectiveness in enhancing patient identification, engagement in care, retention in care programs, treatment adherence, and clinical outcomes. Such a synthesis is needed, given the complexity of implementing models of care delivery that integrate HIV and mental health services as this requires multidisciplinary and inter-professional collaboration, coordination and communication. To address this gap, we systematically reviewed quantitative and qualitative studies describing and evaluating programs or services that seek to integrate HIV and mental health services in adult populations, reporting outcomes where available, and concluding with recommendations for future research.

## Methods

This review was developed according to the PRISMA guidelines (Moher *et al*. 2009) and is one element of a larger systematic review on integration of HIV and non-communicable diseases. Drawing on the definitions proposed by Briggs, Atun, and Legido-Quigley (Groene and Garcia-Barbero 2001, Atun *et al*. 2010a, Briggs and Garner 2006), the concept of integration and its key attributes is described in Box 1 (WHO 2008, Atun *et al*. 2010b).

### Inclusion criteria

We included all quantitative and qualitative studies describing or evaluating a management or organizational change policy or intervention implemented within an existing health system, aiming to integrate HIV and chronic disease care at the service delivery level. To be considered for inclusion for this paper, the studies had to integrate services for one or more mental disorders (*e.g.* depressive, anxiety, substance-related and psychiatric disorders) with HIV, which includes both the integration of mental health services into HIV services, as well as the integration of HIV services into existing mental health services. Services could be provided in health facilities or in the community and include any adult population. We did not exclude reports based on study design; nor did we require them to include outcome measures. We imposed no language, publication date, or publication status restrictions. Conference abstracts were included as this is an important source of unpublished studies.

### Search strategy

The search strategy and terms were developed collaboratively with an information specialist, and were consistent with methods adopted by other authors who have conducted systematic reviews on health services integration (Groene and Garcia-Barbero 2001, Briggs and Garner 2006). We searched the following electronic databases from inception until February 2014: Global Health, Medline and Embase. Key words (MeSH terms) and free text terms were developed for three themes: HIV, integration and chronic diseases and then combined in the search strategy, after which the papers on the integration of HIV and mental health were identified. The search terms used for Medline are shown in Box 2. In addition, we searched the following databases using a simplified search strategy to ensure maximum yield of papers from LMICs: Cochrane library, LILACs, Africa Wide, WHOLIS and abstracts from the International AIDS Society (IAS) Online Resource Library from 2006 to 2015, the HIV Implementers meetings from 2007 to 2012, and international conferences on non-communicable diseases such as the 2014 Annual Meeting of the College on Problems of Drug Dependence and the 2015 Annual Scientific Meeting of the Research Society on Alcoholism, among others. We conducted an updated search until October 2015 using Global Health, Medline and Embase.

### Search and retrieval of studies

Two reviewers independently screened the list of articles obtained following the electronic database search based on title or title and abstract, to identify those meeting the inclusion criteria. If either of the two reviewers considered a study potentially eligible, we retrieved the full text for further assessment. For articles in languages other than English, a reviewer who could read and understand the article assessed it. The reviewers were able to read in Spanish and French. The two reviewers assessed the retrieved full texts independently to assess whether they met the inclusion criteria. Any disagreements were resolved by discussion with a third reviewer.

### Data synthesis

Five reviewers (HLQ, DB, LG, NW and LO) independently extracted data from included studies using standardized forms. Differences in data extraction or interpretation of the studies were resolved by discussion and consensus among the five reviewers and with additional revisions by FLHC, VEH, SEO and FC when there were disagreements among the different pair of reviewers. We extracted data from the results and discussion sections of both quantitative and qualitative studies including information on: (1) study characteristics including study design, setting and sample size, (2) participants characteristics including age, gender, ethnicity and country of origin, (3) integration activities of the intervention, (4) results and type of outcome measure including process and patient outcomes, and (5) the advantages and disadvantages of integration activities as discussed in each study. We conducted a narrative synthesis of the findings.

### Levels of integration

Valentijn's taxonomy of integration which is organized as the dimensions of the Rainbow Model of Integrated Care (Valentijn *et al*. 2013) was used as a framework to categorize papers in the data extraction and synthesis process. Drawing on this analytical framework, we consider integration at the macro level to involve the integration of delivery systems within the HIV, mental health and primary care sectors. We categorised integration at the meso level on two dimensions, i.e. organizational integration and professional integration. Organizational integration involves collaborative networks and relationships between agencies providing HIV, mental health and/or substance abuse services. Professional integration constitutes inter-professional partnerships of a multidisciplinary HIV, mental health and/or substance abuse team based on shared roles, responsibility and accountability reflecting the treatment plans of patients with multiple co-morbidities. At the micro level, clinical integration refers to the coordinated person-centred care in a single process across time, place and discipline, wherein all components of a patient’s care in HIV, mental health and substance abuse are merged into one treatment plan. (Valentijn *et al*. 2015)

### Risk of bias assessment

First, four independent reviewers (LA, NW, DB, LO) assessed risk of bias for papers assigned. Then, a fifth independent reviewer (HLQ) was involved to compare the results and resolve the differences in assessment. The Cochrane risk of bias tool was used to assess randomized control trials (RCT) (Higgins *et al*. 2011) while observational studies was assessed using a proforma with three domains: selection bias, information bias (differential misclassification and non-differential misclassification) and confounding. Each domain was assessed as low, unclear or high. We classified studies that had a low risk of bias in all domains as having a low overall risk of bias. Studies that had a high or a unclear risk of bias in one or more domains were classified as having an overall high or a unclear risk of bias. We evaluated qualitative studies using an adapted version of a checklist used in a previous series of mixed methods systematic reviews (Rees *et al*. 2006, Oliver *et al*. 2008).

## Results

**Figure 1.**
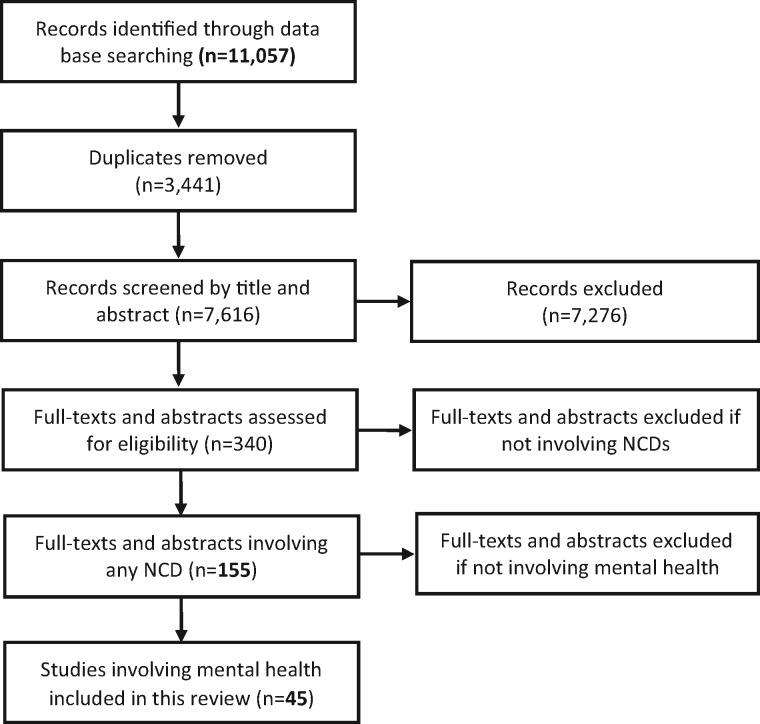
Study flow diagram.

11,057 records were identified during the initial database searches. 7,616 articles, remaining after exclusion of duplicates, were screened by title and abstract for inclusion. 340 full-texts and abstracts were assessed for eligibility and 155 studies were found to include one or more non-communicable disease. For the purpose of this review, we then selected studies addressing HIV and mental health. Forty-five articles met the eligibility criteria for this review (See Figure 1), including 39 full papers and six conference abstracts. All papers reviewed were in English. Due to the heterogeneity in study design, intervention types, participants, and outcomes, we did not conduct a meta-analysis but instead present a summary of the articles, and a synthesis of their results and outcomes where available.

### Characteristics of included studies

**Figure 2.**
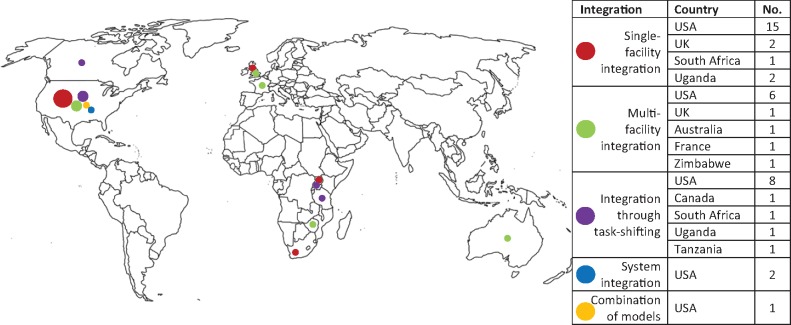
Map by Integration Model.

Of the 45 included studies, 26 of the articles were quantitative, two were qualitative, three were mixed-method studies and 14 were program or model descriptions. Of the 26 quantitative studies, seven were RCTs, five were non randomized intervention studies, five were cohort studies, three were case-series studies, three were cross-sectional studies, and three were retrospective record reviews. Based on the World Bank’s classification of income status, 38 of the 45 studies (84%) were carried out in high-income countries, 32 of which were in the USA, three in the UK, one in Canada, one in Australia and one in France. Two were carried out in an upper middle-income country, South Africa; and five in low-income countries, of which three were in Uganda, one in Zimbabwe and one in Tanzania (See Figure 2 for a geographical representation of the studies by integration models that are described in the following sections).

Five of the 45 papers provided a definition of integration (Table 1). Of the 45 papers, only two studies described integrating HIV services within existing mental health services (Rosenberg *et al*. 2010, Lemmon and Shuff 2001) while in the remaining papers, mental health and/or substance abuse services were integrated within existing HIV services. In 10 of these papers, these services were integrated in primary care settings (Farber *et al*. 2014, Harris and Williams 1995, Winiarski *et al*. 2005, Feingold and Slammon 1993, Wolfe *et al*. 2003, Zaller *et al*. 2007, Wright and Shuff 1995, Esposito-Smythers *et al*. 2014, Nebelkopf and Penagos 2005, Dodds *et al*. 2004).

**Table 1.** Definitions of integration from studies included in the review

Author	Definition of Integration
Lemmon and Shuff 2001	System integration defined as consisting of appropriate referrals and the free-flow exchange of information among service delivery components in mental health care, primary health care and HIV care coordination services
Winiarski *et al.* 2005	Integrated care defined as mental health services provided on-site at the medical clinic
Coleman *et al.* 2012	Collaborative care defined by: (1) its guiding principles as described in The Chronic Care Model (CCM) which includes taking a team-based, patient-centered, collaborative approach that incorporates elements of patient care such as patient registries, patient education, screening or assessment tools, adherence monitoring, and evidence-based treatment guidelines; and (2) the degree of collaboration described as a continuum from less to more collaborative
Weaver *et al.* 2009	The merging of health and medical services conceptualized on a continuum of care ranging from *coordinated*, meaning that care is delivered in different settings with information sharing among programs; to *co-located*, meaning that services are delivered at one location; to *integrated*, meaning that medical and behavioral healthcare components are merged in one treatment plan
Dodds *et al.* 2004	Integrated service systems defined as multifaceted approaches to providing services for patients with complex needs, whereby two or more entities develop linkages to improve outcomes for their clients and combine efforts to serve clients more responsively. This means that providers from multiple disciplines share referrals, collaborate on case planning, and activate the resources of multiple agencies rather than constraining clients to a single agency or program

### Risk of bias assessment

We conducted risk of bias assessments only for papers that evaluated integration of services and reported outcome measures or qualitative results. These included 15 quantitative studies, one mixed-methods study and one qualitative study. Nine studies were assessed to have an overall high risk of bias while seven studies were assessed to have an overall unclear risk of bias, and the qualitative study was assessed as unclear due to missing information. The risk of bias assessment ratings for the 17 studies by domain is shown in Table 5.

### Levels of integration

**Figure 3.**
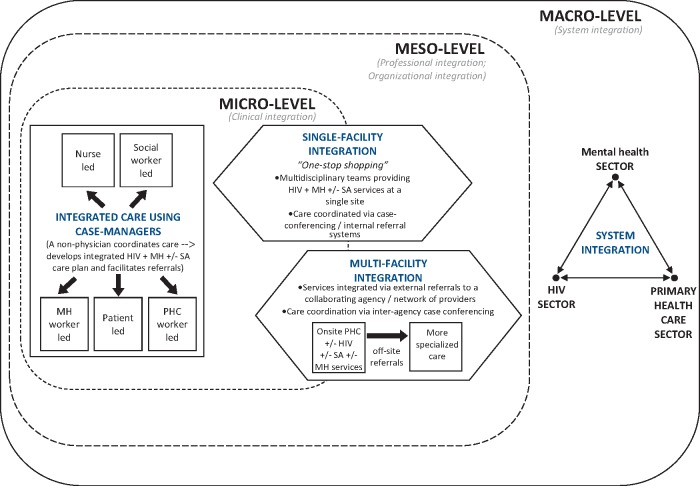
Integration models for HIV, mental health and substance abuse services at the macro, meso, and micro-level.

Of the 45 papers, only two involved integration at the macro level (Wright and Shuff 1995, Lemmon and Shuff 2001). 31 papers involved integration at both the meso and micro level of which two integration models were identified, while the remaining 12 papers involved integration at the micro level only, representing a third integration model in this review (Figure 3 represents the three models graphically by level of integration).

#### Macro-level integration

Both of the macro-level papers were written on the Indiana Integration of Care Project (IICP), a federally-funded project in the USA that integrated mental health services with Indiana’s existing HIV and AIDS service delivery system at the state level (Wright and Shuff 1995, Lemmon and Shuff 2001). One of the papers described the program and the theoretical foundation underlying its conception, and included a cross-sectional baseline analysis of the linkages between community mental health providers with primary care and HIV providers (Wright and Shuff 1995). The other study sought to investigate the effect of mental health centre staff turnover on HIV and AIDS service delivery integration (Lemmon and Shuff 2001).

#### Meso- and micro-level integration

31 papers involved interventions in which integration occurred both at the meso and micro levels. From these papers, two distinct integration models were identified involving integration in a single-facility and integration across multiple facilities. Twelve other papers described interventions that integrated services exclusively at the micro level through the use of case managers, serving as the 3^rd^ distinct model of integration identified. The three models are described below to provide a sense of how HIV and mental health services are integrated at the meso and micro levels.

##### Model 1: single-facility integration

A total of 20 papers involved interventions that integrated services within a single facility. Seventeen were conducted in high-income countries, with 15 in the USA and two in the UK (Surah 2013, Hyam *et al*. 2012), one study was conducted in a middle-income country, South Africa (Jonsson *et al*. 2011), and two studies were conducted in a low-income country, Uganda (Namata Mbogga Mukasa *et al*. 2014, Nakimuli-Mpungu *et al*. 2014). 16 were full papers and four were conference abstracts (Namata Mbogga Mukasa *et al*. 2014, Surah 2013, Cohen *et al*. 2011, Vergara-Rodriguez *et al*. 2012). Of these, there were eight descriptive studies (Feingold and Slammon 1993, Dillard *et al*. 2010, Dodds *et al*. 2004, Harris and Williams 1995, Kobayashi and Standridge 2000, Namata Mbogga Mukasa *et al*. 2014, Wood 2008, Jonsson *et al*. 2011), four cohort studies (Farber *et al*. 2014, Nebelkopf and Penagos 2005, Vergara-Rodriguez *et al*. 2012, Esposito-Smythers *et al*. 2014), three retrospective record reviews (Coleman *et al*. 2012, Cohen *et al*. 2011, Feldman *et al*. 2012), two non-randomized intervention studies (Winiarski *et al*. 2005, Surah 2013), one RCT (Tetrault *et al*. 2012), one mixed-methods study (Hyam *et al*. 2012), and one qualitative study (Nakimuli-Mpungu *et al*. 2014).

In terms of treatment modalities, 6 out of the 20 studies involved interventions that integrated HIV and mental health services (Farber *et al*. 2014, Feldman *et al*. 2012, Harris and Williams 1995, Hyam *et al*. 2012, Feingold and Slammon 1993, Nakimuli-Mpungu *et al*. 2014). In five other studies, the process was part of a larger package of integration with other services, including general primary health care (PHC) (Coleman *et al*. 2012, Winiarski *et al*. 2005) obstetrics and gynaecology (O&G) services (Dodds *et al*. 2004), risk reduction interventions (Namata Mbogga Mukasa *et al*. 2014), TB services (Jonsson *et al*. 2011) and non-communicable disease screening and treatment services (Namata Mbogga Mukasa *et al*. 2014). Three studies involved interventions that integrated HIV, mental health and substance abuse services within a HIV clinic setting (Surah 2013, Vergara-Rodriguez *et al*. 2012, Esposito-Smythers *et al*. 2014) while six others involved integration with primary health care (Cohen *et al*. 2011, Dillard *et al*. 2010, Wood 2008), Hepatitis C treatment (Tetrault *et al*. 2012), risk reduction interventions (Nebelkopf and Penagos 2005) and specialist services (Kobayashi and Standridge 2000) in a single site. Table 2 lists the papers describing this model presented according to treatment modality and setting.

**Table 2.** Single-facility integration

Integration Model	Treatment Modality	Setting	Author and Country	
Single-facility Integration	HIV + Mental Health	Primary care clinic	Farber *et al.* 2014 [USA] Harris and Williams 1995 [USA] Feingold and Slammon 1993 [USA]	3
		AIDS service organization	Feldman *et al.* 2012 [USA]	1
		Sexual health clinic	Hyam *et al.* 2012 [UK]	1
		Trauma clinic	Nakimuli-Mpungu *et al.* 2014 [Uganda]	1
	HIV + Mental Health + Other services	Primary care clinic	Winiarski *et al.* 2005 [USA] Dodds *et al.* 2004 [USA]	2
		HIV clinic	Coleman *et al.* 2012 [USA] Namata Mbogga Mukasa *et al.* 2014 [Uganda] Jonsson *et al.* 2011 [South Africa]	3
	HIV + Mental Health + Substance Abuse	HIV clinic	Esposito-Smythers *et al.* 2014 [USA] Surah 2013 [UK] Vergara-Rodriguez *et al.* 2012 [USA]	3
	HIV + Mental Health + Substance Abuse + Other services	Primary care clinic	Nebelkopf and Penagos 2005 [USA]	1
		HIV clinic	Wood 2008 [USA] Tetrault *et al.* 2012 [USA] Kobayashi and Standridge 2000 [USA]	3
		Substance abuse treatment site	Dillard *et al.* 2010 [USA]	1
		Residential facility	Cohen *et al.* 2011 [USA]	1

The single-facility integration model, otherwise known as ‘one-stop shopping‘, allows patients to access a variety of services at a single site. Four studies described that care coordination was implemented through regular case conferences bringing together members of the multidisciplinary team (Nebelkopf and Penagos 2005, Winiarski *et al*. 2005, Wood 2008, Kobayashi and Standridge 2000), while in one case, individual discussions, voicemails and shared medical notes were used as additional means to coordinate care (Winiarski *et al*. 2005). One study described an internal referral system to facilitate interdepartmental care coordination (Feldman *et al*. 2012). In another study conducted in the USA, there were also joint consultations involving HIV primary care, and mental health providers, in addition to case discussions and referrals. In this study, the degree of collaboration varied according to the patients’ needs along the care continuum (Feingold and Slammon 1993). The single-facility integration model involved activities both at the meso- and micro-levels, with professional integration based on multidisciplinary inter-professional partnerships and clinical integration based on patient-centered case conferencing and joint consultations.

The heterogeneity of the study locations indicates that this model of integration has been implemented in a wide range of different settings; although most commonly, services were integrated within primary care clinics (Farber *et al*. 2014, Harris and Williams 1995, Feingold and Slammon 1993, Winiarski *et al*. 2005, Dodds *et al*. 2004) or in a HIV clinic (Coleman *et al*. 2012, Namata Mbogga Mukasa *et al*. 2014, Jonsson *et al*. 2011, Esposito-Smythers *et al*. 2014, Surah 2013, Vergara-Rodriguez *et al*. 2012, Wood 2008, Tetrault *et al*. 2012, Kobayashi and Standridge 2000). In six of the studies in these settings, mental health services comprised of specialized psychiatric liaison services or consultative treatment (Coleman *et al*. 2012, Harris and Williams 1995, Kobayashi and Standridge 2000, Surah 2013, Hyam *et al*. 2012, Vergara-Rodriguez *et al*. 2012). In some of the studies, integration was implemented through a specific treatment program. Examples include: a measurement-based approach to depression care (Coleman *et al*. 2012), and cognitive behavioural therapy and contingency management measures (Esposito-Smythers *et al*. 2014, Nakimuli-Mpungu *et al*. 2014).

The advantages of the single-facility integration model were discussed in some of the papers. From a provider’s perspective, single-site integration of services is perceived to enhance communication between providers, and reduce scheduling and coordination time (Coleman *et al*. 2012, Dillard *et al*. 2010). The involvement of a multidisciplinary team on site also increases the likelihood that the overall needs of a patient with dual or triple-diagnoses are considered within the treatment plan and competing priorities are addressed and minimised, reducing the occurrence of contradictory treatment demands (Dillard *et al*. 2010). From a patient’s perspective, this model of integration reduced physical barriers to access, including transportation which often hampers continuous access to care, and other practical challenges facing those with mental or physical impairment (Dillard *et al*. 2010). Integration with primary health care or with other services, was also reported to improve confidentiality that might be breached when someone is seen attending a specialist mental health or HIV facility, reducing stigma and alleviating some of the anxiety among patients seeking care. (Coleman *et al*. 2012, Harris and Williams 1995, Wood 2008, Dillard *et al*. 2010). On the contrary however, it may be more difficult to implement single-site integration in smaller cities or rural areas where there is a lack of resources. Providing a full continuum of care within one facility may not be practical or cost-effective for patients with multiple co-morbidities, as they may need a more comprehensive or specialised range of healthcare services (Wood 2008).

##### Model 2: multi-facility integration

In 10 of the studies, services were integrated via inter-agency collaborations or mechanisms for external referrals to an intermediary: a collaborating agency or a collaborative network of providers. Nine of the studies were conducted in a high-income country, six of which in the USA (Curran *et al*. 2011, Daughters *et al*. 2010, Woods *et al*. 1998, Wood 2008, Taylor 2005, Rosenberg *et al*. 2010), one in Australia (Sternhell *et al*. 2012), one in France (Leclerc *et al*. 2005) and one in UK (McCarthy *et al*. 1992); one study was conducted in a low-income country, Zimbabwe (Duffy *et al*. 2014). Nine studies were reported in full papers and one was a conference abstract (Duffy *et al*. 2014). Of these, there were four descriptive studies (Woods *et al*. 1998, Wood and Austin 2009, Sternhell *et al*. 2012, Taylor 2005), two case-series (Daughters *et al*. 2010, Leclerc *et al*. 2005), two RCTs (Curran *et al*. 2011, Rosenberg *et al*. 2010), one non-randomized intervention study (McCarthy *et al*. 1992) and one mixed-methods study (Duffy *et al*. 2014).

In terms of treatment modalities, 2 of the 10 studies involved interventions that integrated HIV and mental health services (Curran *et al*. 2011, Duffy *et al*. 2014) while in one other study, these services were also integrated with Hepatitis C treatment (Sternhell *et al*. 2012). Three studies involved interventions that integrated HIV, mental health and substance abuse services (Daughters *et al*. 2010, Leclerc *et al*. 2005, Woods *et al*. 1998) while three other study interventions integrated these services along with primary health care (Wood and Austin 2009), genitourinary services (McCarthy *et al*. 1992), hepatitis treatment (Taylor 2005), and risk reduction services (Rosenberg *et al*. 2010). Table 3 lists the papers in which integration involved multiple facilities, presented according to treatment modality and description of referral channels.

**Table 3.** Multi-facility integration

Integration Model	Treatment Modality	Description of Referrals	Author and Country	
Multi-site Integration (off-site referrals)	HIV + Mental Health	Off-site referrals to mental health specialists	Curran *et al.* 2011 [USA]	1
		Referrals between community/traditional medicine practitioners and public health facilities	Duffy *et al.* 2014 [Zimbabwe]	1
	HIV + Mental Health + Other services	Off-site referrals to mental health specialists	Sternhell *et al.* 2012 [Australia]	1
	HIV + Mental Health + Substance Abuse	Off-site referrals to mental health specialists	Daughters *et al.* 2010 [USA] Leclerc *et al.* 2005 [France]	2
		Off-site referrals for substance abuse services	Woods *et al.* 1998 [USA]	1
	HIV + Mental Health + Substance Abuse + Other services	Off-site referrals for HIV specialist services	McCarthy *et al.* 1992 [UK]	1
		Inter-agency referrals and care coordination within a collaborative network of specialist organizations	Wood and Austin 2009 [USA]	1
		Off-site referrals for medical services	Rosenberg *et al.* 2010 [USA]	1
		Off-site referrals to a mental health agency	Taylor 2005 [USA]	1

In most of the studies, integration of services generally occurred via established referral systems between facilities or agencies that provide separate services (Duffy *et al*. 2014, Sternhell *et al*. 2012, Rosenberg *et al*. 2010, Daughters *et al*. 2010, McCarthy *et al*. 1992, Taylor 2005). In four of these studies however, off-site referrals were made only when the patient required more specialized mental health or HIV services (Daughters *et al*. 2010, Curran *et al*. 2011, McCarthy *et al*. 1992, Sternhell *et al*. 2012). For example, in one of the interventions that combined a brief behavioural activation approach and cognitive behavioural approach to treat depression and improve HIV medication adherence, patients were only referred for psychiatric treatment at a different facility when they were diagnosed with a psychiatric condition (Daughters *et al*. 2010). In two other studies, providers communicated through a network of agencies, and referrals were conducted via linkages between agencies within the established network (Woods *et al*. 1998, Wood and Austin 2009). In one of these studies, regular inter-agency case-conferences were also organized to coordinate patient care (Wood and Austin 2009). The multi-facility integration model involves integration at both meso- and micro-levels. Professional and organizational integration is achieved through collaboration of different specialized agencies mediated via collaborative networks and referral mechanisms, while clinical integration occurred through inter-agency case conferences and joint consultations.

In this model of integration, a facility may offer a range of integrated services co-located at one site and coordinate with other agencies and professionals for more specialized services. From a provider’s perspective, the advantage of a multi-facility integration model such as this lies in the practicality and cost-effectiveness of offering a comprehensive range of services to patients with complex needs. One study described a community-based multiservice organization in the USA, which had a HIV and AIDS intensive case management and coordination unit, but reported that it was not feasible to provide the entire continuum of care on-site as the complexity of the patients’ medical and social problems demanded a more comprehensive package of services. In this case, it seemed more practical to create a collaborative network of agencies (Wood and Austin 2009). In another study, however, splitting services over different sites was presumed to create barriers, as patients accessing different medical providers received fragmented, inconsistent, and poorly coordinated care (Daughters *et al*. 2010).

##### Model 3: integration through care-coordination using case managers

In 12 of the studies, integration of services involved the use of a non-physician, such as a nurse or a social worker, acting as a case manager responsible for developing an integrated treatment care plan and facilitating referrals. Nine of the studies were conducted in a high-income country, of which eight were in the US (Andersen *et al*. 2003, Sullivan *et al*. 2015, Adams *et al*. 2011, Wolfe *et al*. 2003, Zaller *et al*. 2007, Adams *et al*. 2012b, Sacks *et al*. 2011, Bouis *et al*. 2007) and one in Canada (Husbands *et al*. 2007); one was conducted in a middle-income country, South Africa (Andersen 2012); and two were conducted in low-income countries, in Uganda (Odokonyero *et al*. 2015) and in Tanzania (Adams *et al*. 2012a). Eleven studies were reported in full papers and one was a conference abstract (Andersen 2012). Of these, there were two descriptive studies (Andersen *et al*. 2003, Zaller *et al*. 2007), three RCTs (Adams *et al*. 2012b, Husbands *et al*. 2007, Sacks *et al*. 2011), two non-randomized intervention studies (Bouis *et al*. 2007, Adams *et al*. 2011), one cohort study (Adams *et al*. 2012a), one case-series (Odokonyero *et al*. 2015), one cross-sectional study (Wolfe *et al*. 2003), one mixed-methods study (Andersen 2012) and one qualitative study (Sullivan *et al*. 2015).

In terms of treatment modalities, eight out of the 12 studies involved interventions that integrated HIV and mental health services (Andersen *et al*. 2003, Sullivan *et al*. 2015, Odokonyero *et al*. 2015, Adams *et al*. 2012a, Andersen 2012, Husbands *et al*. 2007, Adams *et al*. 2011, Adams *et al*. 2012b) while four studies involved interventions that integrated HIV, mental health and substance abuse services (Wolfe *et al*. 2003, Zaller *et al*. 2007, Sacks *et al*. 2011, Bouis *et al*. 2007). Table 4 lists the papers that described interventions which had case managers who integrated services for patients through a care plan.

**Table 4.** Integration through care-coordination using case managers

Integration Model	Treatment Modality	Person Coordinating Care	Author and Country	
Integration through care-coordination via the use of case managers	HIV + Mental Health	Nurse	Andersen *et al.* 2003 [USA] Sullivan *et al.* 2015 [USA] Odokonyero *et al.* 2015 [Uganda] Adams *et al.* 2012a [Tanzania]	4
		Primary care staff	Andersen 2012 [South Africa]	1
		Social worker	Husbands *et al.* 2007 [Canada] Adams *et al.* 2011 [USA]	2
		Depression-care manager	Adams *et al.* 2012b [USA]	1
	HIV + Mental Health + Substance Abuse	Primary care staff	Wolfe *et al.* 2003 [USA] Zaller *et al.* 2007 [USA]	2
		Patient/Client	Sacks *et al.* 2011 [USA]	1
		Social worker	Bouis *et al.* 2007 [USA]	1

Out of the 12 studies, four described integrated care led by a nurse (Andersen *et al*. 2003, Sullivan *et al*. 2015, Odokonyero *et al*. 2015, Adams *et al*. 2012a), three described integrated care led by primary care staff (Andersen 2012, Wolfe *et al*. 2003, Zaller *et al*. 2007), three led by a social worker (Husbands *et al*. 2007, Adams *et al*. 2011, Bouis *et al*. 2007), one led by a depression-care manager (Adams *et al*. 2012b) and one that was integrated by the patient (Sacks *et al*. 2011). In most of the studies, the case manager was responsible for providing or facilitating integrated care by linking patients and assisting them to access necessary services as part of an integrated treatment plan (Sullivan *et al*. 2015, Andersen *et al*. 2003, Husbands *et al*. 2007, Zaller *et al*. 2007, Bouis *et al*. 2007). In some instances, the development of the treatment plan involved the collaboration between the care coordinator and patient or care providers (Andersen *et al*. 2003, Zaller *et al*. 2007, Bouis *et al*. 2007). In two studies, the nurse or primary care staff was also responsible for conducting screening for depression (Odokonyero *et al*. 2015), other mental health issues or substance abuse (Wolfe *et al*. 2003). In one study, the patients themselves were taught to coordinate service components of a modified therapeutic community aftercare program and integrate their own treatment. Through various self-help strategies and support groups, patients were educated on how to navigate services and were provided tools to manage and monitor vital elements of their treatment progress. Such client-level integration was perceived to be effective in bridging the gaps in care coordination and empowering clients to track and adhere to the key elements of their treatment plan (Sacks *et al*. 2011).

The use of an algorithm-based tool for prescription and medication management by a nurse or depression-care manager was described in three studies (Odokonyero *et al*. 2015, Adams *et al*. 2012a, Adams *et al*. 2012b), of which two discussed it as part of a measurement-based approach to depression care involving the use of routine symptom measurement to inform treatment planning (Adams *et al*. 2012a, Adams *et al*. 2012b). In all three studies, the care manager was supported or supervised by a psychiatrist. It was propounded that this model of integration could help address the problem of under-diagnosis of depression in PLHIVs, account for antidepressant-antiretroviral interactions, and facilitate quality antidepressant management within HIV care (Adams *et al*. 2012b).

As described in one study, the nurse coordinating the care played a key role in helping patients access resources and providing psychosocial support and education on how to interact with doctors, and served as a source for patients to seek clarification when they were unsure about the information given by providers (Sullivan *et al*. 2015). Another perceived advantage of this integration model was its ability to promote continuity of care for patients as they relate to a single case manager. Yet to achieve these advantages, much effort is required on the part of the case manager to initiate collaborations between providers, which can be hindered by the competing priorities of the various providers with a different disciplinary orientation. As such, appropriate professional training of case managers is essential (Bouis *et al*. 2007).

### Measures of effectiveness of integration

Seventeen studies involved evaluation of one or more measures of effectiveness of an integrated program, intervention, model or approach. We define *patient outcomes* as changes in the health status of the patients or their knowledge, attitudes and behaviours, while *service delivery outcomes* are defined as measures that reflect the effectiveness of the processes involved and delivery of integrated services. The 17 studies described at least one measure of effectiveness in either of these types of outcome, none of which reported long-term impacts on morbidity or mortality indicators (See Table 5 for the results of the studies that evaluated integration including a summary of the patient and process outcomes).

**Table 5.** Results of the studies reporting patient and process outcomes

Integration Model	Study	Objective	Setting and sample size	Study design	Patient outcomes (clinical and behavioral outcomes)	Process outcomes (processes, cost)	Risk of bias assessment
Integration at Macro-level	(Lemmon and Shuff 2001)	To investigate the effect of mental health centre staff (MHCS) turn-over on HIV and AIDS service delivery integration across three service delivery components: primary health care, mental health services, and HIV and AIDS dedicated care coordination	Indiana, US. *n* = 51 MHCS from 17 mental health centres that participated in the Indiana Integration of Care Program (IICP).	Cross-sectional	–	Higher staff turnover rates had no negative impact on integration, with the exception of within-centre services. Mental health service providers are aware of who network providers are, but integration broke down at a level of implementation in terms of contacts, exchange of information and referrals	Unclear risk of selection and performance bias; High risk of detection bias; Low risk of attrition and reporting bias
Single-facility Integration	(Coleman *et al.* 2012)	To assess effectiveness of an integrated, measurement-based approach to depression care where psychiatric consultation service was offered and linked with primary health care	Boston, US. Tertiary hospital. *n* = 124 People living with HIV and AIDS.	Retrospective record review—cohort (pre and post treatment analyses)	Reduction in depression scores from an average BDI-II score of 23 to 15.7 (*P=*0.00001) Reduction in HIV RNA from 14.1 K to 4 K copies/mL, (*P = *0.003) Increase in CD4 count of 518 to 592 (*P = *0.001)	More patients prescribed antidepressants and stimulants post vs. pre treatment	High risk of selection bias; Unclear risk of non-deferential bias
	(Cohen *et al.* 2011)	To assess an integrated care program co-locating medical, mental health, substance abuse and social services	US. Transition centre (TC). *n* = 96 triply-diagnosed patients.	Retrospective record review—cohort (pre and post enrollment analyses)	Increase in virologic control in percentage of months in care from 9% to 42% (*P < *0.0001) Before TC, CD4 declined an average 19 cells/yr; after enrollment, CD4 increased an average 34 cells/yr (*P < *0.0001)	Patients engaged in care 95% of the time after enrollment in TC as compared to 81% prior to enrollment (*P < *0.0001)	Unclear quality as results are presented in abstract format
	(Winiarski *et al.* 2005)	To evaluate the effectiveness of a HIV mental health program integrated with primary care that emphasized cultural responsiveness	Inner-city of South Bronx, US. Health clinic. *n* = 147 HIV patients.	Intervention study (non-randomized)	Reduction in mental health problems [*F* (1, 58) = 8.22, *P < *0.01] Reduction in HIV symptoms [*F* (1, 34) = 8.67, *P < *0.01] Decrease in alcohol use [*F* (1, 37) = 15.21, *P < *0.01] and cocaine use [*F* (1, 79) = 7.03, *P < *0.01] Improved social functioning [*F* (1, 83) = 4.35, *P < *0.05]	Treatment group used mental health services at a higher rate than comparison group	Unclear risk of selection and attrition bias; Low risk of reporting bias
	(Feldman *et al.* 2012)	To evaluate the Rapid Response System (a set of operating procedures designed to facilitate interdepartmental linkage of clients to mental health evaluation) in an AIDS service organization	New York, US. AIDS service organization. *n* = 314 clients of the AIDS organization.	Retrospective record review (cohort)	–	Of the 281 clients who scheduled an appointment for an evaluation to initiate MH services, 64% completed the evaluation Decrease in likelihood of completing the mental health evaluation as the number of days between Rapid Response System contact and date of evaluation appointment increased (AOR=0.84, CI = 0.78, 0.92)	Unclear quality as results are presented in abstract format
	(Farber *et al.* 2014)	To examine perceived stigma among HIV patients before and after participation in a mental health program co-located within two urban community-based HIV primary care settings	Southeastern US. Community-based primary care setting. *n* = 48 HIV patients.	Cohort (pre and post intervention)	Reductions in self-reported perceived HIV stigma 3 months in three dimensions: distancing (*t*=4.01, *P = *0.000, d = 0.43); blaming (*t*=2.79, *P = *0.008, d = 0.35); and discrimination (*t*=2.90, *P = *0.006, d = 0.42)	–	Unclear risk of selection bias, High risk of non-differential bias
	(Vergara-Rodriguez *et al.* 2012)	To assess the effects of an integrated treatment program (H-Star) offering co-located substance abuse and psychiatric evaluation and treatment	Chicago, US. Co-located psychiatric and substance abuse service site. *n* = 123 dual-diagnosis patients.	Cohort (assessments at baseline and 6 months)	Statistically significant reduction in use of alcohol, heroin and cocaine at 6 months	Of 136 participants, 75 (55.1%) had psychiatric evaluations; 53 (70.7%) received medication management	Unclear quality as results are presented in abstract format
	(Surah 2013)	To evaluate integrated care versus standard care offered in a psychiatric-led clinic	Ireland, UK. In-reach HIV clinic. *n* = 37 HIV infected injecting drug users.	Intervention study (non-randomized)	Clinical outcomes improved significantly with the introduction of the intervention Substance and alcohol misuse, HRQOL and Hospital Anxiety Depression scale data were not significantly different between cases and controls over 1 year	–	Unclear quality as results are presented in abstract format
Multi-facility Integration	(Rosenberg *et al.* 2010)	To assess the STIRR intervention designed to facilitate integrated infectious disease programming in mental health settings, and to increase acceptance of such services among clients (STIRR = screening and testing for HIV and hepatitis, immunization for hepatitis A and B, risk-reduction counseling, referral and support for medical care)	Baltimore, US. Community mental health services sites. *n* = 236 clients with co-occurring mental illness and substance use disorders, of whom 19 had HIV.	RCT	Intervention group more likely to reduce substance abuse Intervention group showed no reduction in risk behavior and were not more likely to be referred to care (81% vs. 75%) and showed no increase in HIV knowledge	Intervention group had high levels (over 80%) of participation and acceptance of core services Intervention group more likely to be tested for hepatitis B and C; immunized for hepatitis A and B; Intervention costs → $541 per client	Low risk of selection, detection, reporting and attrition bias; High risk of performance bias
	(Daughters *et al.* 2010)	To examine the integration of a combined depression and HIV medication adherence treatment program	Washington DC, US. Residential substance abuse treatment centre. *n* = 3 case series.	Case series	Improvements in rates of depression, initiation of a HAART regimen, and HIV medication adherence across all cases Increase in behavioral activation and environmental reward in two out of three cases	–	Descriptive case study, not assessed
	(Duffy *et al.* 2014)	To examine feasibility of implementing a Stepped-Care Model between community as well as traditional medicine practitioners and health facilities (referrals using standard operating procedures and trainer manuals)	Zimbabwe. *n* = 30 staff.	Mixed-methods (qualitative followed by a survey)	–	80-100% of eligible clients received referrals for higher level mental health and/or psychosocial services Linked traditional medicine practitioners into the health system and motivated clients to complete referrals Increased awareness of and comfort discussing mental health problems with clients 80% of respondents/trained staff (*n*=30) agreed that stigma was reduced in facilities following training	Unclear quality as results are presented in abstract format
Integration through care-coordination using case managers	(Adams *et al.* 2011)	To test the feasibility and appropriateness of a collaborative depression case model whereby care was coordinated by a social worker	North Carolina, US. Outpatient infectious diseases clinic. *n* = 13.	Intervention study (non-randomized)	Depression scores measured using PHQ-9 decreased from 18.33±6.06 to 11.44±7.91 (t-2.73, df = 8, *P = *0.03)	–	High risk of selection and non-differential bias
	(Adams *et al.* 2012a)	To test the feasibility of a task-shifting model of measurement based depression care in a HIV clinic	Tanzania. Outpatient HIV care and treatment centre. *n* = 20 HIV patients.	Cohort (assessments at baseline, 4 weeks and 12 weeks)	Depression scores measured using PHQ-9 decreased from 19.76 at baseline to 8.12 at week-12 (*t*=19.62, df = 16, *P < *0.001)	–	High risk of selection and non-differential bias; Low risk of differential bias; Unclear risk of confounding
	(Husbands *et al.* 2007)	To assess a case management approach used to support integrated services developed in a service organization to support HIV patients	Toronto, Canada. AIDS Service Organization. *n* = 79 HIV patients.	RCT	Those who were very depressed benefited the most from case management which markedly improved their physical, social and mental health functioning, and reduced their risk behaviors	Case management participants’ use of community services was associated with an economically important, though not statistically significant, $3,300 per person per annum lower expenditure for the use of all direct health and social services	High risk of selection, detection and attrition bias; Unclear risk of performance and reporting bias
	(Andersen 2012)	To assess the feasibility and usefulness of implementing a cognitive behavioral based intervention for treatment of adherence and depression	Cape Town, South Africa. Community health clinic and MSF clinic. *n* = 14 HIV patients with major depressive disorder.	Qualitative	Reported reduction in depressive symptoms, global distress and level of impairment	–	Unclear quality as results are presented in abstract format
	(Sacks *et al.* 2011)	To evaluate an integrated therapeutic community aftercare program in which clients were taught to coordinate service components (HIV + mental health + substance abuse) and integrate their own treatment	Philadelphia, US. *n* = 76 triply diagnosed patients.	RCT	Moderate treatment effects in terms of substance use and mental health favouring participants in intervention group in the High propensity stratum (Hedge’s g -0.34, *P < *0.002)	–	High risk of performance, detection and attrition bias; Low risk of selection and reporting bias
Combination of three models at different sites	(Weaver *et al.* 2009)	To evaluate the cost-effectiveness of integrated HIV primary care, mental health, and substance abuse services among triply diagnosed patients	US. Multisite. *n* = 431 triply diagnosed patients.	RCT (cost-effectiveness assessment)	–	Decrease in total average monthly cost of health services intervention group: $3,235 to $3,052; control group: $3,556 to $3,271 not statistically significant Significant decrease in percentage attributable to hospital care (intervention group: 37% at baseline to 28%, *P < *0.001; control group: 32% to 29%, *P < *0.001	Unclear risk of selection and performance bias; High risk of detection bias; Low risk of attrition and reporting bias

#### Macro-level integration

Of the 17 studies, one study evaluated integration at the macro level, investigating the effect of staff turnover on HIV and AIDS service delivery integration across three service components comprising of primary health care, mental health services, and HIV and AIDS dedicated care coordination. This cross-sectional study surveyed a sample of 51 staff from 17 mental health centres and found that staff turnover rates did not negatively impact integration, except for within-centre services, i.e. when HIV was integrated within the mental health system itself [t(15) = +0.05, *P >* 0.05]. The overall risk of bias was unclear, although the study identified some important challenges in the implementation of integration relating to poor communication and information sharing within centre, which can lead to a breakdown of referral patterns and limit access to quality patient care (Lemmon and Shuff 2001).

#### Meso- and micro-level integration

Among the 15 studies that reported one or more measures of effectiveness of integration at the meso and micro levels, seven studies involved single-site integration, three studies involved multi-facility integration and five studies involved integrating services through a case-manager. One study in particular, involved all three models of integration. This was an RCT in the US that assessed the cost-effectiveness of integrated HIV primary care, mental health and substance abuse services for triply diagnosed patients where integration was across four different sites using single-site multidisciplinary case management, off-site referrals, and care coordinated by an adherence counsellor or nurse. Patients were randomly assigned to the intervention group (*n* = 232) receiving integrated care, or the control group (*n* = 199) who received care-as-usual. At the end of the 12-month trial, the total average monthly cost of health services decreased from US$3,235 to US$3,052 in the intervention group and US$3,556 to US$3,271 in the control group, but the decreases were not statistically significant. The percentage attributable to hospital care in both groups decreased, but there were no significant differences between them in annual cost of health services and quality of life. The overall risk of bias for this study was unclear (Weaver *et al*. 2009).

##### Model 1: single-facility integration

Among the seven studies, some assessed specific approaches like the measurement-based approach to depression care (Coleman *et al*. 2012) while others evaluated operating systems to facilitate inter-organizational referrals (Feldman *et al*. 2012). Four studies compared outcomes before and after intervention (Coleman *et al*. 2012, Cohen *et al*. 2011, Farber *et al*. 2014, Vergara-Rodriguez *et al*. 2012) and one retrospectively reviewed clinic data of a patient cohort on completion of referrals (Feldman *et al*. 2012). Collectively, these studies reported improvements in clinical outcomes of HIV and mental health disorders, reduction in substance use behaviours and stigma, improvements in social functioning, and higher patient engagement in care, although the overall risks of bias of the studies were high or unclear. The evidence substantiating these reported outcomes are specified in Table 5.

Two other studies of integration within a single-site were non-randomized intervention studies (Winiarski *et al*. 2005, Surah 2013). In a study conducted in the US, 47 PLHIVs in the treatment group who received integrated mental health, HIV and primary care services designed to be culturally responsive and co-located within a single site; were compared to a control group of 100 PLHIVs who had access only to usual care, which included mental health services that were non HIV-specific and not co-located with primary care. Utilization rates were higher among the treatment group and this was associated with fewer mental health problems [*F* (1, 58) = 8.22, *P <* 0.01], HIV-related physical symptoms [*F* (1, 34) = 8.67, *P <* 0.01], alcohol [*F* (1, 37) = 15.21, *P <* 0.01] and cocaine use [*F* (1, 79) = 7.03, *P <* 0.01], and improvements in social functioning [*F* (1, 83) = 4.35, *P <* 0.05]. The overall risk of bias for this study was unclear (Winiarski *et al*. 2005). The other non-randomized intervention study evaluated integrated care versus standard care among HIV-infected intravenous drug users seeking services at a HIV clinic with psychiatry-led addiction services in Ireland. Thirty clients were recruited to the intervention group and 26 to the control group. Clinical outcomes improved significantly among the intervention group, although there were no significant differences in health-related quality of life (HRQOL), anxiety, depression and substance misuse between the groups (Surah 2013). The risk of bias for this study was unclear as information was presented in an abstract only.

##### Model 2: multi-facility integration

Three studies assessing programs involving multi-facility integration reported outcomes reflecting one or more measures of effectiveness. One study examined the integration of a combined depression and HIV medication adherence program of three case series which reported improvements in depression rates, initiation of HAART and medication adherence (Daughters *et al*. 2010). Another study using mixed-methodology sought to determine the feasibility of a Stepped-Care Model integrating services between community, traditional medicine practitioners and health facilities using standard operating procedures and trainer manuals. The survey in this study presented a high percentage of successful referrals (80–100%), as well as increased awareness and reduced stigma among healthcare personnel in treating patients with co-morbidities (Duffy *et al*. 2014). These were not assessed for risks of bias.

The third study is an RCT that assessed the STIRR intervention (Screening and Testing for HIV, Immunization against hepatitis A and B, Risk-reduction counselling, and Referral and support for medical care). This intervention sought to facilitate integrated infectious disease programs in mental health settings and increase acceptance of such services among clients. The trial recruited 236 dually diagnosed clients receiving services at a community mental health centre and randomly assigned them to the STIRR intervention (*n* = 118) or the control group (*n* = 118). The control group received enhanced usual treatment, which included information on blood-borne diseases, information on local community health sources for blood testing, immunization against hepatitis A and B, and treatment as needed. Subjects randomized to STIRR had high levels (over 80%) of participation and acceptance of core services and were more likely to be tested for hepatitis B and C (88% vs. 14% at 6 months); immunized for hepatitis A and B (76% vs. 5% at 6 months); have an increase in their hepatitis knowledge (*F =* 15.68, *P <*0.001) and reduce their substance abuse (*F =* 4.54, *P =* 0.34). However, they had no reduction in risk behaviour, were no more likely to be referred to care (81 vs. 75%) and gained no increase in HIV knowledge. The risk of bias was generally low in all regards except for the potential performance bias as subjects and researchers were not blinded. (Rosenberg *et al*. 2010).

##### Model 3: integration through care-coordination using case managers

Five studies assessing programs involving integration by a case manager had reported outcomes reflecting one or more measures of effectiveness, of which three were feasibility studies. One adopted a qualitative design in assessing a cognitive behavioural based intervention in an integrated program and reported reductions in depressive symptoms, global distress and level of impairments, although risks of bias could not be assessed (Andersen 2012). The other cohort study sought to test the feasibility of a task-shifting model of measurement-based depression care, reporting a reduction in depression score measured with PHQ-9 from 19.76 at baseline to 8.12 at week-12 (t = 19.62, df = 16, *P <* 0.001) (Adams *et al*. 2012a). Finally, the third study—a non-randomized intervention study, evaluated the feasibility of a collaborative depression care model using social workers to coordinate care and found a decrease in depression scores measured with PHQ-9 from 18.33 ± 6.06 to 11.44 ± 7.91 (t-2.73, df = 8, *P =* 0.03) (Adams *et al*. 2011). However, the risk of selection and non-differential bias were rated as high.

There were two RCTs associated with this integration model. One sought to evaluate an integrated therapeutic community aftercare program for triply diagnosed individuals in Philadelphia, US. Forty-two (55%) subjects were assigned to the intervention group who received integrated care and 34 (45%) to the control group who received standard aftercare services. The intervention consisted of health and self-management groups, peer-support groups, self-help groups, individual case assistance and family support groups designed to ensure treatment continuity and to assist patients’ transition to more independent functioning in the community. Among the group of participants who had greater psychological and physical health at baseline, those in the intervention group had greater overall improvements in their mental health and substance use than those in the control group (Sacks *et al*. 2011).

The other study was an RCT in Canada that assessed a case management approach used to support integrated services implemented in a service organization located in Toronto to support PLHIVs. The study sample comprised 79 patients randomized to either the intervention or control group, although the potential for selection bias was evident. The intervention group undertook self-directed use of services facilitated by a social worker who would assist the patient in accessing support, while the control group received care-as-usual comprising only self-directed use of services. Those with more severe depression benefited the most from case management which had a positive effect on their physical, social and mental well-being, as well as on their risk behaviours. Additionally, participants’ use of community services was associated with a lower expenditure for all direct health and social services (Husbands *et al*. 2007).

## Discussion

This review brings together evidence on different models that have sought to integrate HIV and mental health services, ranging from integration within a single facility to multi-facility integration and integrated care coordinated by a non-physician case manager. The treatment modalities integrated within each model differed; some were more complex than others, especially those that included substance abuse and other types of services. This, coupled with the difference in setting – *e.g.* rich and poor countries, varied packages of care, and the broad spectrum of degree of integration, i.e. less to more integrated – affirms that it is not possible to draw firm conclusions about the effectiveness of models. However, some tentative deductions can be drawn on the potential advantages and disadvantages of the differing integration activities and strategies within each model from a patient and provider perspective (Table 6).

**Table 6.** Potential advantages and disadvantages of each model.

Models of integration	Potential Advantages		Potential Disadvantages	
	Patient-perspective	Provider-perspective	Patient-perspective	Provider-perspective
Model 1: Single-facility integration	Increases access to care Increases screening and testing for HIV/mental health/substance abuse problems Reduces physical barriers (*e.g.* transportation) to access Increases comfort and safety of patients Increases confidentiality Lesser risk of stigma (less likely for public to spot if the health center offers a wide range of services) Normalizes anxiety of patients seeking care Patients engage more in care than those who receive services accessible by off-site referrals	Enhances communication between providers Reduces scheduling and coordination time Ensures all needs of patients are considered in treatment planning No competing priorities in the treatment planning for dual or triple diagnosis patients Reduces staff splitting Places appropriate responsibility on each professional in the multidisciplinary team to assist patients in prioritizing treatment requirements Cost-effective in larger urban areas with plentiful resources and higher concentrations of PLHIVs	Sharing common spaces within a facility may lead to stigma and a lack of privacy, serving as a barrier to accessing services	More difficult to employ in smaller cities and rural areas due to a lack of resources Providing a full continuum of care on-site may not be cost-effective as dual or triple diagnosis patients may need a more comprehensive set of healthcare services Difficult in settings where there is a lack of mental health specialists Requires a wide range of supply of medicines and goods
Model 2: Multi-facility integration via inter-agency collaboration or off-site referrals	Allows for patient choice and preference for specialized care	Practical and cost-effective when offering a comprehensive and diverse range of services to patients with complex needs (not possible to cover in one single facility)	Barriers to accessing services, *e.g.* increased patient cost for transport and for attending multiple facilities Failed referrals Difficulties monitoring outcomes	Fragmented, inconsistent and poorly coordinated care Process of forming collaborations is time consuming and requires commitment of agency resources Agencies may have differing missions, clinical orientations, or legal needs
Model 3: Integration through the use of case manager	Supports continuity of care Case managers serve as a focal point for clarification and education Case managers serve as social support Trust relationship is built between case manager and patient	Addresses under-diagnosis and under-treatment of mental health issues among HIV patients Accounts for critical antidepressant-antiretroviral interactions	Clients can become dependent on their case-manager, reduced personal responsibility over their individual care plan Loss of doctor-based care (which is perceived as the best care)	Case-managers are challenged with the task of fostering collaboration between providers which may be hindered by the differences in clinical orientation and competing priorities Requires comprehensive training of case managers

As expected, single-site integration is advantageous where there are already different providers working under one roof. The heterogeneity of the study locations indicates that this model of integration can be implemented in a wide range of settings. Single-site integration can increase access to services by reducing the inconvenience, additional costs and physical barriers that (often vulnerable) patients may encounter (Coleman *et al*. 2012, Dillard *et al*. 2010, Wood 2008). However, providing a full continuum of care for patients with dual or triple diagnoses who may need a more comprehensive range of services, can be costly and impractical. Multi-facility integration through a collaborative network of specialized agencies may be more effective when the treatment needs of a patient with multiple co-morbidities are beyond what can be provided within a single facility (Wood and Austin 2009). In some instances, integration via a system that facilitates rapid referrals may be more appropriate, particularly when a patient requires very specialized care or when few mental health specialists are available (Wood 2008). However, the degree to which services are integrated via referral mechanisms can be examined, as integration activities can range from mere referrals between services (least integrated), to having more formalized referral systems and linkages organized within a pre-established network of agencies that coordinate care via inter-agency case conferencing (more integrated). Effective referral systems supported by appropriate coordination mechanisms may be needed to prevent fragmented and poorly coordinated care in multi-facility integration.

Integrated care coordinated by a case manager can enable continuity of care for patients. However, this requires that these cadres have adequate training in the separate areas of HIV, mental health and substance abuse and are well supported, if they are to coordinate care effectively. This model of integration may be adapted in LMICs given the limited resources, scarcity and poor distribution of mental health specialists. While studies from LMICs is limited, the available evidence seem to show that task-shifting mechanisms may be feasible and beneficial through the use of less specialised personnel such as nurses, medical assistants and ‘expert clients’ who can be trained in detecting, screening and managing psychological conditions under the supervision of a psychiatrist (Odokonyero *et al*. 2015, Adams *et al*. 2012a). In LMICs, the integration of services may also need to consider alternative providers such as traditional medicine practitioners (Duffy *et al*. 2014). Active screening can be possible via the innovative use of data collection tools implemented within existing HIV facilities to effectively identify patients with potential symptoms of mental illness (Namata Mbogga Mukasa *et al*. 2014). Additionally, consideration of the social and cultural context in which patients conceptualize their beliefs and understanding of mental illness and treatment are likewise important in the development of integrated services of HIV and mental health (Nakimuli-Mpungu *et al*. 2014).

We also identified some novel approaches to integration, wherein patients were taught how to coordinate service components within their own treatment plan via self-management and support groups designed to educate patients on how to navigate services and use self-help tools to monitor vital elements of their treatment progress (Sacks *et al*. 2011). This model not only bridges the gaps in care coordination, but also engages with patients and enables them to take personal responsibility for decision-making and management of their own care. There is evidence that empowering the patients can increase the likelihood of positive treatment outcomes while reducing the burden on healthcare resources and capacities (Swendeman *et al*. 2009). However, regardless of which model is adopted, the context in which it is implemented must be taken into account, including factors such as resource availability and distribution, as well as the patient’s specialized needs and where they are on the continuum of care: diagnosis, initiation of treatment, care for additional morbidities etc. Culture, institutional and social norms, as well as patient and family preferences are likely to be important in determining whether the patient will be motivated to play an active role in their own treatment (Martin *et al*. 2005).

Very few papers in this review defined integration. When defined, the term was commonly used interchangeably with collaborative or coordinated care to describe similar models of service delivery. Definitions varied greatly, from describing the term as simply as a co-location of services to more comprehensive descriptions of coordinated care along a continuum that included referrals and linkages of services via inter-agency collaborations. This is expected considering the complexity and multi-dimensionality of integrating multiple treatment modalities in striving to deliver quality and cost-effective care to patients with dual and triple diagnoses. A previous systematic review on measurements of integrated healthcare delivery supports this notion that despite the vast literature on the subject, there is no consistent definition or fully-developed concept of service integration (Strandberg-Larsen and Krasnik 2009). The lack of conceptual clarity challenges the systematic understanding of integrated care and its attributes, which could hamper the design, delivery, management and evaluation of integrated programs (Valentijn *et al*. 2013). A clearer construct of the complex phenomenon of integrated care at the outset can help to guide empirical research and validate the evaluation outcomes of integration, thus allowing an accurate assessment of whether activities designed truly reflect an integration of services that is cost-effective, and that ultimately improves patient outcomes. Additionally, conceptual clarity on what integration should or should not be, and the attributes that underlie the integrating activities could help interpret evidence better on the value of the various integration models.

### Study strengths and limitations

A strength of this review was the use of a wide range of databases and conference archives to increase the number of papers from LMICs for inclusion, although studies identified from the search were mostly from high income countries, particularly the USA which could be due to publication bias. There are few real world initiatives that are evaluated, and it is also possible that studies with null findings are less likely to be published. Although conference archives were searched as a source of unpublished studies, conclusions could not be drawn on effect sizes and risks of biases of these interventions due to the limited information provided in these abstracts. Similarly, a majority of the papers included in this review were descriptive. While these provided useful insights on the approaches and strategies adopted in integrating HIV and mental health services, we could not infer the effectiveness of the various interventions described. In total, there were 17 studies that reported measures of effectiveness on integration, of which only four were RCTs. These studies were of variable methodological quality, a majority of which had an overall high or unclear risk of bias.

### Implications for research

This review reveals that much of the research on integrated HIV and mental health care has described small-scale interventions or specific treatment approaches that involve some degree of integration activities at the meso and micro levels. Evidence on the effectiveness of systemic approaches to the integration of HIV, mental health and substance abuse services at the macro-level is clearly lacking. Further research is necessary to evaluate functional approaches to integration that engage with the financing, information systems, and management modalities of service delivery within health systems. There is also a need for evidence on strategies that could facilitate the normative underpinnings of integrated care, including shared-values, culture and goals across individuals, professionals, organizations and systems (Valentijn *et al*. 2013).

Additionally, none of the papers reviewed reported long-term outcomes or impacts relevant to HIV or substance abuse, such as mortality. The longest period over which outcomes were measured was 6 months. Also, none of the papers compared outcomes or cost between different models of integration. This exemplifies the need for higher quality and robustly designed studies that seek to evaluate and compare integration models in terms of their long-term impact on patient outcomes and system-level outcomes. These may include mortality and morbidity indicators relevant to the disease progression of HIV and mental disorders; as well as the reporting of service coverage outcomes, institutional-based outcomes, and cost-effectiveness of real-world interventions. The incorporation of evaluative elements in study designs is also necessary to identify stronger causal linkages between intervention components and desired outcomes.

Given the varying needs of patients with HIV along the care continuum, there is a need for more evaluation of interventions that seek to integrate services at the pre-antiretroviral and end-of-life phases in HIV care. In this review, we found no studies that explored interventions at these periods. Additionally, very few studies described the integration of HIV screening or care into existing mental health services. As described at the beginning of this paper, mental health conditions are known to precipitate HIV transmission behaviours and affect antiretroviral therapy adherence. Therefore, further research is needed to address the under-diagnosis and under-treatment of HIV infection among patients with serious mental illnesses. Additionally, none of the studies reviewed involved integration of HIV and mental health services within antenatal care programs. Further research is necessary since previous studies have identified psychiatric symptoms – particularly depression, as a common condition among pregnant women with HIV globally (Kapetanovic *et al*. 2014).

While this review sought to include papers in languages other than English and studies conducted in different geographical regions, only seven papers were identified from LMIC countries within Eastern and Southern Africa, which has the greatest burden of the AIDS epidemic. A previous systematic review revealed that the majority of HIV and AIDS and mental health studies in sub-Saharan Africa focused on mental health-related HIV risk behaviours, HIV in psychiatric populations, and mental illness in HIV-positive populations (Breuer *et al*. 2011). As such, more research is needed on how best to integrate HIV and mental health services in this region. Importantly, there were no studies from Asia or Latin America, signifying the need for more research in these regions too.

Findings from the intervention studies provide some evidence on the effectiveness of integration activities in yielding positive patient outcomes, particularly on improvements in mental health, HIV symptoms, social well-being and substance misuse. However, differences between intervention and control groups were not statistically significant for some of these measures in a number of the studies, especially in regards to patient’s improvement in quality-of-life and in one study, the annual cost-savings of health services (Weaver *et al*. 2009). It is nevertheless imperative to be cognizant of the diversity in integration approaches adopted and varying methodologies across the studies. Overall, the heterogeneity in integration activities, patient populations, study designs and analysis strategies make it difficult to draw any firm conclusions for policy, beyond the finding that integration, which *a priori* seems a sensible goal to pursue, has been shown to be associated with some improved outcomes in diverse settings. However, given the scope for publication bias noted above, the implementation should, where possible, be accompanied by rigorous evaluation methodologies. While it is highly beneficial to measure process outcomes to identify strategies in overcoming integration barriers and the contextual drivers for successful integration, evaluation should move beyond the mere measurement of process indicators to address more importantly, the short and long-term patient outcomes, which is fundamentally the primary aim of integration itself.

## Conclusions

This review identified a diversity of integration models combining HIV and mental health services at the meso and micro levels, each with its respective advantages and disadvantages from the patient and providers’ perspective. These provide insight into the principles that could underpin the development and implementation of integrated care models for HIV and mental health services. Firstly, single-site integration augments multidisciplinary coordination while reducing access barriers, but can be difficult to implement when a fuller continuum of specialized care involving multiple treatment modalities is needed particularly in low-resource settings. Secondly, multi-facility integration may comprehensively serve multi-morbid patients, but appropriate coordination and referral mechanisms are crucial to prevent fragmented care. Thirdly, active case management by non-clinicians offers considerable potential especially in low resource settings with shortages of mental health specialists, although appropriate training and support is essential. Finally, involving the patients not just as service users but also as active partners in improving integration within the treatment process, is a promising approach. While the current body of evidence on integration of HIV and mental health services from this review presents several benefits encompassing a myriad of positive patient and service delivery outcomes, the imperative for higher quality and robustly designed evaluative studies is evident, particularly in LMICs. As national planners and policy makers consider new ways of financing, implementing, managing and evaluating integrated care for HIV and mental health services, the evidence reviewed here can contribute to this process.

Box 1 Definition of integration

Managerial or operational changes to health systems to bring together inputs, delivery, management and organization of particular service functions as a means of improving coverage, access, quality, acceptability and (cost)-effectiveness. This may include:

- Service integration: interventions that combine ‘different packages of services’
- Integration of service delivery points which include health units of any type for *e.g.* primary care settings, hospitals, residential settings, service organizations etc.
- Integration at different levels of service delivery: macro-, meso-, micro-levels
- Process modifications to facilitate integration for *e.g.* referral and linkage mechanisms or standard operating procedures
- Introduction of technologies aimed at aiding integration
- Integration of management decisions

Box 2 Search Strategy used for Medline, Embase and Global Health via Ovid (adapted to only include mental health and substance abuse terms)

Database: Embase <1980 to October 2015>, Global Health <1910 to October 2015>, Ovid MEDLINE(R) <1946 to October Week 4 2015>

1. ((vertical or horizontal or integrat* or coordinat* or co-ordinat* or link*) and (program* or care or service*)).mp. or delivery of health care, integrated/or primary healthcare/
2. exp HIV infections/or HIV.mp. or Human immunodeficiency virus.mp. or “HIV/aids”.mp.
3. (All introduced in a separate line) chronic disease/or long-term care/or ((chronic* or persistent or long* term or ongoing or degenerative) adj3 (disease* or disab* or ill* or condition* or health condition* or medical condition*)).tw. or long* term care.tw. or (non-communicable disease* or NCD).tw. or exp neurodegenerative diseases/or (neurodegenerative or Huntington* disease or Parkinson* disease or amyotrophic lateral sclerosis or motor neuron disease).tw. or exp cerebrovascular disorders/or (cerebrovascular disease* or cerebrovascular disorder* or brain ischaemia or cerebral infarction or carotid artery disease* or stroke).tw. or exp dementia/or (dementia or alzheimer*).tw. or exp depression/or exp mental disorder/or (mental health or depression).tw. or exp alcoholism/or alcohol*.tw. or exp substance-related disorders or substance misuse.tw.
4. 1 and 2 and 3
